# Deuterated Arachidonic Acid Ameliorates Lipopolysaccharide-Induced Lung Damage in Mice

**DOI:** 10.3390/antiox11040681

**Published:** 2022-03-31

**Authors:** Alla Y. Molchanova, Svetlana N. Rjabceva, Tigran B. Melik-Kasumov, Nikolay B. Pestov, Plamena R. Angelova, Vadim V. Shmanai, Olga L. Sharko, Andrei V. Bekish, Genevieve James, Hui Gyu Park, Irina A. Udalova, J. Thomas Brenna, Mikhail S. Shchepinov

**Affiliations:** 1 Institute of Physiology, National Academy of Sciences of Belarus, Academicheskaya 28, 220072 Minsk, Belarus; alla@fizio.bas-net.by (A.Y.M.); sveta.rjabceva@tut.by (S.N.R.); biblio@bas-net.by (T.B.M.-K.); 2 Laboratory of Tick-Borne Encephalitis and Other Viral Encephalitides, Chumakov Federal Scientific Center for Research and Development of Immune-and-Biological Products of Russian Academy of Sciences, Poselok Instituta Poliomielita 8 bd 17, Poselenie Moskovsky, 108819 Moscow, Russia; 3 Group of Cross-Linking Enzymes, Shemyakin-Ovchinnikov Institute of Bioorganic Chemistry, Miklukho-Maklaya 16/10, 117997 Moscow, Russia; 4 Queen Square Institute of Neurology, University College London, Queen Square, London WC1N 3BG, UK; p.stroh@ucl.ac.uk; 5 Institute of Physical Organic Chemistry, National Academy of Sciences of Belarus, Surganova 13, 220072 Minsk, Belarus; v.shmanai@gmail.com (V.V.S.); olsharko@gmail.com (O.L.S.); andreibekish@yahoo.com (A.V.B.); 6 Departments of Pediatrics, of Chemistry, and of Nutrition, Dell Pediatric Research Institute, University of Texas at Austin, 1400 Barbara Jordan Blvd, Austin, TX 78723, USA; gejames@utexas.edu (G.J.); hi.park@austin.utexas.edu (H.G.P.); tbrenna@utexas.edu (J.T.B.); 7 The Kennedy Institute of Rheumatology, University of Oxford, Oxford OX1 2JD, UK; irina.udalova@kennedy.ox.ac.uk; 8 Retrotope, Inc., Los Altos, CA 94022, USA

**Keywords:** lung, acute respiratory distress syndrome, arachidonic acid, D-PUFA, eicosanoids, isotope effect

## Abstract

Arachidonic acid (ARA) is a major component of lipid bilayers as well as the key substrate for the eicosanoid cascades. ARA is readily oxidized, and its non-enzymatic and enzymatic oxidation products induce inflammatory responses in nearly all tissues, including lung tissues. Deuteration at bis-allylic positions substantially decreases the overall rate of ARA oxidation when hydrogen abstraction is an initiating event. To compare the effects of dosing of arachidonic acid (H-ARA) and its bis-allylic hexadeuterated form (D-ARA) on lungs in conventionally healthy mice and in an acute lung injury model, mice were dosed with H-ARA or D-ARA for six weeks through dietary supplementation and then challenged with intranasal lipopolysaccharide (LPS) for subsequent analysis of bronchoalveolar lavage fluid and lung tissue. Dosing on D-ARA resulted in successful incorporation of D-ARA into various tissues. D-ARA significantly reduced LPS-induced adverse effects on alveolar septal thickness and the bronchoalveolar area. Oral deuterated ARA is taken up efficiently and protects against adverse LPS-induced pathology. This suggests novel therapeutic avenues for reducing lung damage during severe infections and other pathological conditions with inflammation in the pulmonary system and other inflammatory diseases.

## 1. Introduction

Excessive inflammatory damage to lung tissue during bacterial or viral infections occurs with a dysregulated eicosanoid axis and hindered repair of complex components of cellular membranes and secreted surfactant proteolipids [1]. Acute respiratory distress syndrome (ARDS) is an aggravating condition; ARDS is associated with aging and contributes to mortality in as many as 40% of deaths. ARDS is often caused by bacterial or viral pneumonia. In rodents, ARDS is usually modeled as acute lung injury (ALI) by instillation of live bacteria (*S. pneumoniae* or *P. aeruginosa)* or non-specific damaging factors such as ozone. Bacterial ALI can be modeled by a sterile single dose challenge with *E. coli* lipopolysaccharide (LPS).

Arachidonic acid (ARA, 20:4(n-6)), a metabolically essential polyunsaturated fatty acid (PUFA), is a vital building block for biological membranes. ARA is also the main substrate for several enzymes, including cyclooxygenase/prostaglandin-endoperoxide synthase (in most mammals—two or three isoforms, in both mice and humans—COX1 and COX2), arachidonate lipoxygenase (LOX/ALOX, six isoforms in humans, seven in mice), and the P450 cytochromes. These enzymes generate multiple pro-inflammatory and pro-thrombotic eicosanoid mediators, such as prostaglandins, leukotrienes, and thromboxanes, which initiate further inflammatory cascades. Inflammation increases the level of reactive oxygen species (ROS) that initiate the non-enzymatic chain reaction of ARA peroxidation (LPO) as well as enzymatic oxidation, leading to multiple toxic products.

**Figure 1:**
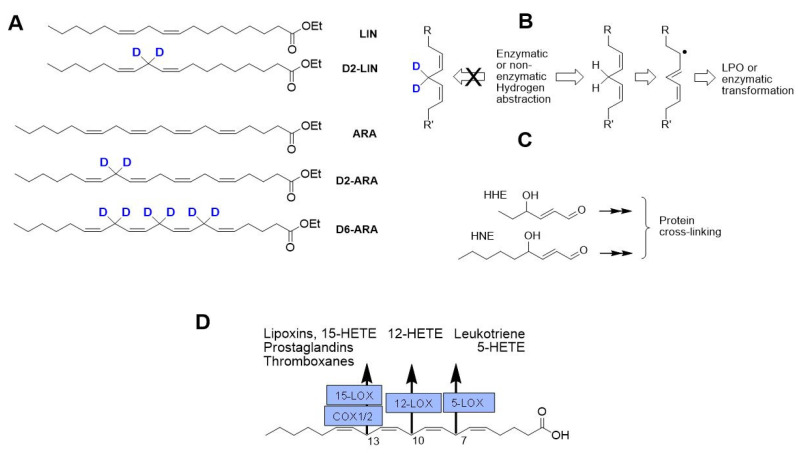
(**A**) PUFAs used in this study. LIN, linoleic acid; D2-LIN, 11,11,D2-linoleic acid; ARA, arachidonic acid; D2-ARA, 13,13-D2-arachidonic acid (a product of in vivo enzymatic elongation/extension of D2-LIN); D6-ARA, 7,7,10,10,13,13-D6-arachidonic acid. (**B**) Hydrogen abstraction of a bis-allylic hydrogen, the key rate-limiting step of PUFA oxidation (both enzymatic and LPO), is inhibited by deuteration. (**C**) Multiple products of non-enzymatic LPO include reactive carbonyls such as HHE and HNE, which can covalently cross-link biomolecules. (**D**) Numerous products of enzymatic ARA oxidation are mostly pro-inflammatory and pro-thrombotic.

Both enzymatic and non-enzymatic oxidation occurs at any of the three bis-allylic sites (7,10,13) within the ARA molecule (Figure 1). Hydrogen abstraction, a C-H bond cleavage, is the key rate-limiting step for both types of transformation. ARA oxidation plays a pivotal role in multiple aspects of the cytokine storm syndrome and thrombotic complications, especially in acute conditions during bacterial and viral infections [2]. COX and LOX inhibitors can be employed, with unpredictable efficacy, to down-regulate this process. Our novel approach relies on deuteration of the three oxidation-prone sites within the ARA molecule to substantially slow down the rate-limiting step of oxidation (both enzymatic and non-enzymatic) via the isotope effect (IE) [2,3], reducing LPO and inflammation. Here we studied the effects of dietary arachidonic acid (H-ARA) or its hexadeuterated form (D-ARA) on the lungs and gastrointestinal tract in healthy and LPS-treated mice. 

## 2. Materials and Methods

### 2.1. D6-ARA and D2-Lin Synthesis

11,11-D2-linoleic acid ethyl ester was prepared through a 5-step synthesis, with the key step involving a copper-catalyzed coupling of a deuterated alkyne bromide with a 10-carbon-long terminal alkyne with subsequent hydrogenation of the triple bonds [4]. The hexadeuterated ethyl arachidonate was synthesized catalytically from a natural, non-deuterated ethyl ester of arachidonic acid, as previously described [5]. 

### 2.2. In Vivo Model and Sampling Protocols

In the preliminary study, mice (six weeks old, both genders) received LIN or D-LIN once per day via oral gavage, 50 μL per mouse. Upon completion of the PUFA treatment, mice were intranasally administered with 1 mg/kg of LPS [6] and sacrificed 24 h later for lung histology.

The arachidonic acid-containing mouse diet was from Research Diets (New Brunswick, NJ, USA), based on fat-free AIN-93G. Fat was added at 11.3% by weight total fat and 0.25% either normal H-ARA or D-ARA, respectively (Table 1). The H-ARA diet served as the control to test whether the bis-allylic deuteriums had an effect independent of ARA.

Male and female BALB/c mice were provided with standard environmental conditions, i.e., 22 ± 1 °C, 55 ± 5% humidity, and a 12 h light/dark cycle with free food and water access. At the age of six weeks, animals were split into 8 groups (*n* = 160 total, 20 of each gender) and were fed ad libitum on the diet containing either H-ARA or D-ARA for 2, 4, 6 weeks at ca. 5 g per mouse per day for the duration of the study. The other two groups of animals (*n* = 40, 20 of each gender) received neither PUFA-enriched diets nor LPS served as controls. Body weight and food intake were measured once a week. After completing the appropriate diet course, half of the mice in each group (10 females and 10 males) (i) underwent bronchoalveolar lavage (BAL) to enable cytological smears and (ii) were subjected to tissue harvesting after humane euthanasia with a lethal dose of sodium thiopental (100 mg/kg intravenously). On the ninth week of this diet course, the other half of the group was administered 1 mg/kg of intranasal (IN) LPS from *E. coli* O111:B4 (Sigma/Aldrich, Santa Clara, CA, USA) 24 h prior to BAL and subsequent euthanasia. For washout experiments, C57BL/6 mice were fed for more than 8 weeks: at the ninth week, 40 D-ARA fed animals (20 males, 20 females) were switched to the H-ARA diet. 

### 2.3. Bronchoalveolar Lavage (BAL) Fluid Collection

BAL was performed as described elsewhere [7]. Briefly, lungs were lavaged three times using three aliquots of 1 mL 0.15 M NaCl under deep anesthesia (sodium thiopental). Each lavage consisted of slow infusion and gentle aspiration of saline via a tracheal cannula. Three recovered lavages were combined. The samples were centrifuged for 7 min at 400× *g* and 4 °C in a microcentrifuge with a F241.5P rotor (Beckman Coulter, Brea, CA, USA). The supernatants were collected and stored frozen at −80 °C for no longer than one month before further analysis.

### 2.4. Histological Analysis

Lungs were fixed with 4% formaldehyde (freshly prepared from paraformaldehyde), paraffin-embedded and cut into 2–4 μm sections. For routine histology, all fixed tissue sections were stained with hematoxylin and eosin (H & E). All slides were evaluated at 10 fields each using a Genetic Pro Bino light microscope (A) and photographed with a digital camera (Delta Optical, North Little Rock, AR, USA) or Aperio AT2 histological scanner (Leica, Wetzlar, Germany). The severity of lung injury was scored according to lung pathological changes, including interalveolar and perivascular hemorrhages, perivascular and peribronchial infiltration, perivascular edema, interalveolar septal thickness, and emphysematous transformations. Emphysema was quantified based on the measurement of the mean linear intercept (MLI) and destructive index (DI). Briefly, the MLI was measured by dividing the length of a line drawn across the lung section by a total number of intercepts counted within this line [8]. The DI was calculated by dividing the defined destructive alveoli by the total number of alveoli. Lung tissue samples were additionally stained with a commercially available Martius Scarlet Blue (MSB) stain kit (Avantik, Pine Brook, NJ, USA) for fibrin visualization. Homogeneous fibrin masses of yellow, pink, or red color present in the lung blood vessels were interpreted as thrombotic masses. 

### 2.5. Cytokine Measurements with Enzyme-Linked Immunosorbent Assay (ELISA) 

Cell-free BAL fluid supernatants as well as colon tissue samples were analyzed using a commercially available Mouse IL-1β ELISA Kit (R & D systems, Lot P265854, Minneapolis, MN, USA) and a Biotek ELx-808 microplate reader (Biotek, Winooski, VT, USA) according to the instructions issued by the manufacturer. 

### 2.6. ARA Isotopic Analysis (GC-MS/MS)

The relative ratio of H-ARA to D-ARA was determined by gas chromatography (GC) coupled to chemical ionization mass spectrometry as described in part elsewhere [9]. Briefly, freshly harvested samples were homogenized and extracted by the Bligh and Dyer method, placed in tubes, solvent-evaporated, blanketed with dry nitrogen, sealed, and shipped to Austin (TX) for further sample preparation and analysis. Dried samples were converted to fatty acid methyl esters (FAME) by a one-step method of hydrolysis and methylation. Solvent-mediated (SM) chemical ionization was performed with CH_3_CN as reagent using a Shimadzu GCMS-TQ8040 (Columbia, MD, USA) instrument with a BPX70 capillary column (25 m × 0.22 mm × 0.25 μm; Trajan, Pflugerville, TX, USA). D-ARA was chromatographically separated from H-ARA. Total ion chromatograms were used to integrate peak areas. 

### 2.7. Statistical Analysis

Experimental data were processed using the Statistica 10.0 software package. Data are shown in graphs as medians and interquartile ranges (Me; Q25%; Q75%). For intergroup comparison, the nonparametric Kruskal–Wallis test was used, with adjustment for multiple comparisons. Differences were considered statistically significant with a *p*-value equal to or less than 0.05. 

## 3. Results

### 3.1. Effect of D-Linoleic Acid (D2-LIN) on the Lungs in LPS-Treated Mice

**Figure 2:**
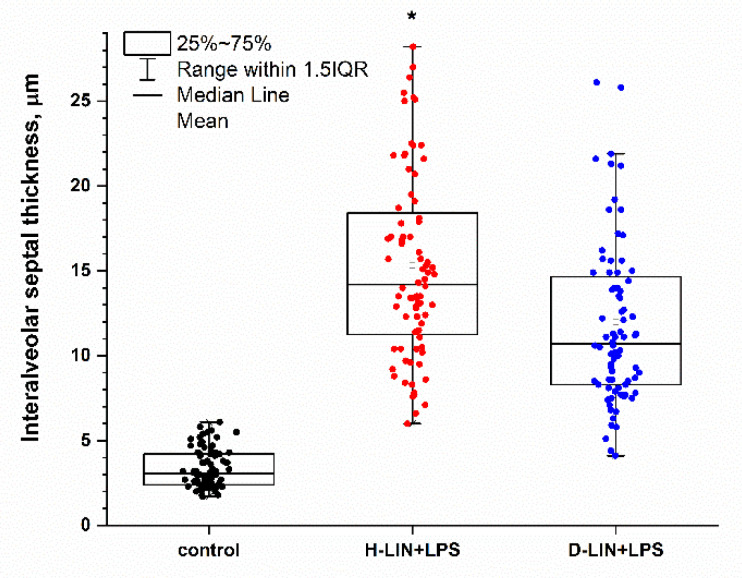
Interalveolar septal thickness (µM) of BALB/c mice after 6 weeks of H- or D-forms of linoleic acid treatment (H-LIN and D2-LIN) followed by single intranasal administration of lipopolysaccharide. *—*p* ≤ 0.05, compared H-Lin versus D2-Lin mice.

In our first experiment, we evaluated the effects of linoleic acid (LIN) supplementation on interalveolar septal thickness in LPS-treated mice. The results (Figure 2) demonstrated a small but statistically significant decrease in interalveolar septal thickness in D2-LIN mice when compared to the H-LIN group. This could be explained by a more pronounced hyperergic response of lungs to H-LIN compared with D2-LIN. LPS treatment revealed signs of hyperplasia of the bronchial epithelium and its hypersecretion, focal perivascular and peribronchial lymphoid infiltrates, together with the presence of serous edema. Histologically, this effect was accompanied by inflammatory cell infiltration through the alveolar walls. 

Since LIN is partially converted to D2-ARA by the action of fatty acid desaturases 1 and 2 (FADS1 and FADS2) and by fatty acid elongase 5 (ELOVL5) [2], we hypothesized that the aforementioned effects of D-PUFA can be attributed to partial deuteration of ARA, and that direct D6-ARA supplementation would be much more efficient than that with D2-LIN.

### 3.2. Pharmacokinetic Aspects of Dietary Supplementation of D-ARA in Healthy Mice

**Figure 3:**
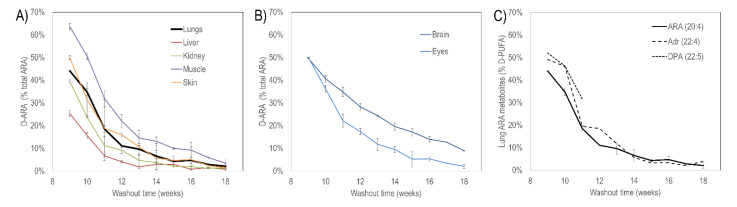
D-ARA as a percentage of all ARA at eight weeks dosing and washout over the subsequent nine weeks with H-ARA feeding. (**A**) Visceral organs and skin. Lung incorporated about 45% D-ARA. (**B**) Neural tissue: whole brain and whole eyes. Both incorporated about 50% D-ARA. Washout from eyes was more rapid than from the brain. (**C**) Lung D-ARA and conversion to longer chain PUFA adrenic acid (22:4(n-6)) by elongation and docosapentaenoic acid (DPA, 22:5(n-3)) by desaturation. All PUFAs followed similar washout kinetics.

As the first step to test the aforementioned hypothesis, we studied the metabolic effect of such a dietary supplementation. We determined the actual content of D-ARA and then performed a washout experiment after a long period of supplementation by switching from the D-ARA to the H-ARA diet followed by MS analysis of several mouse tissues in the ARA diet-fed mice. Nine weeks of D-ARA dosing results in more than 50% incorporation of the deuterated form (25%–65% of all ARA as D-ARA depending on tissue) (Figure 3). 

Brain fatty acid metabolism tends to be distinct from that in the visceral tissues, including overall turnover. Neural PUFAs are uniquely unsaturated, with ARA and omega-3 docosahexaenoic acid (DHA) together comprising more than 25% of all brain fatty acids, while linoleic acid is below 1%. Lung fatty acids are similar to those in other visceral tissues.

### 3.3. Effect of D-ARA on the Response to LPS

H- and D-ARA were well tolerated without apparent adverse reactions. Consumption of the diets for the duration of the experiment did not lead to significant changes in the level of IL-1β in BAL fluid or large intestine homogenates of conventionally healthy mice, regardless of gender (Supplementary Data). Macroscopically, the lungs of all groups were normal at the time of euthanasia. 

**Figure 4:**
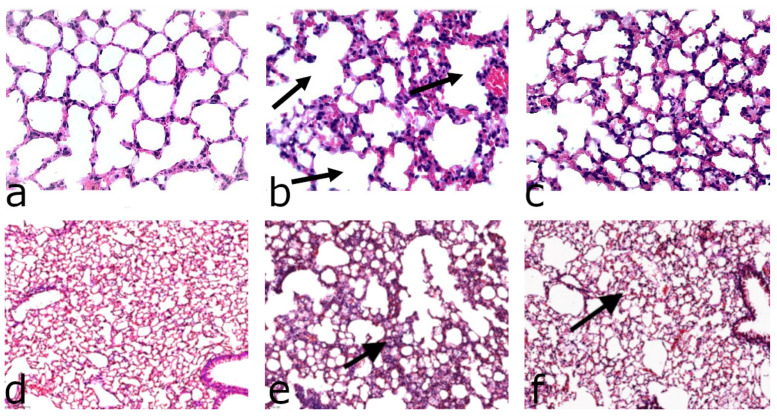
Histological evaluation of the effect of intranasal administration of lipopolysaccharide on the lungs of H-ARA- and D-ARA-treated male mice. **Top**: increased alveolar septal thickness in LPS-treated mice: (**a**) Norm; (**b**) LPS-treated on H-ARA diet; (**c**) LPS-treated on D-ARA diet. Arrows—emphysematous area. **Bottom**: lower magnification sections with emphysematous areas in LPS-treated mice: (**d**) Norm; (**e**) LPS-treated on H-ARA diet; (**f**) treated on D-ARA diet. Arrows—increased thickness of alveolar septa in LPS-treated mice. Bar, 100 μM.

We challenged mice fed ARA-diets for 6 weeks using LPS treatment. Twenty-four hours after exposure to LPS, the lung tissue showed intense perivascular, peribronchial, and septal inflammatory infiltration, with a predominance of lymphocytes and macrophages. Perivascular edema, signs of thrombosis, interalveolar septal thickening, irregular distribution of air spaces, and focal areas of alveolar hemorrhage were also observed (Figure 4). We preferred intranasal administration to the more extreme intratracheal route that induces IL-1β, which in turn amplifies inflammation, making it difficult to distinguish between the early and late consequences of an LPS challenge. Indeed, 24 h after intranasal administration of LPS at a dose of 1 mg/kg, there was no significant increase in the IL-1β content in BAL fluid or colon homogenates compared to animals not treated with LPS, regardless of type or duration of the diets (Supplementary Data). Intranasal LPS 24 h post administration increased neutrophils in BAL fluid in all treated groups, regardless of gender or form of ARA diets; however, no statistically significant differences between the effects of H- and D-ARA were noted for any of the analyzed inflammatory cell types, regardless of gender or LPS treatment (Supplementary Materials). 

**Figure 5:**
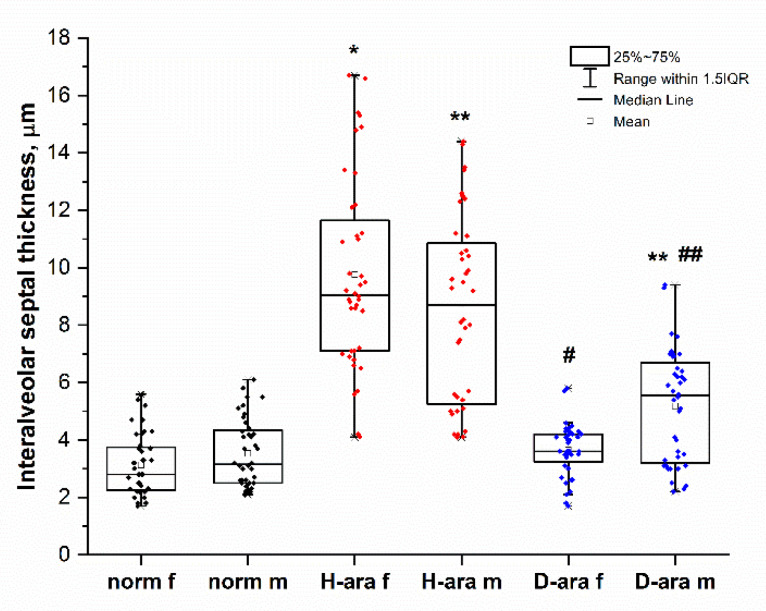
Interalveolar septal thickness (µM) after a 6-week course of H- or D-ARA by single intranasal administration of LPS. Norm, male and female mice without LPS treatment. *—*p* ≤ 0.05, H-ARA female mice versus control females; **—*p* ≤ 0.05, H-ARA male mice versus control males; #—*p* ≤ 0.05, H-ARA female mice versus D-ARA females; ##—*p* ≤ 0.05, H-ARA male mice versus D-ARA males.

In mice on 6 weeks of dietary D-ARA followed by single intranasal administration of LPS, the thickness of the interalveolar septa was significantly decreased compared to the control H-ARA diet (Figure 4, top); interalveolar septal thickness is likely indicative of more edema and greater inflammatory severity accompanied by immune cell infiltration. LPS-induced perivascular edema as measured by interalveolar septal thickness in mice consuming the control H-ARA diet was mitigated in the male D-ARA group and was almost negligible in D-ARA females (Figure 5). The magnitude of the intranasal LPS effect in mice on our custom control H-ARA diets (Figure 5) is similar to that in mice on our basal laboratory chow (Supplementary Materials), although this should be interpreted with caution. Despite the long-established influence of specific PUFA levels on inflammation, the influence of the dietary lipid components (n-3 to n-6 ratios, PUFAs versus long chain PUFAs, total fat level) on LPS-induced damage in mice has never been systematically investigated. Most reports ignore lipid composition, simply stating that a “chow” was used. This makes it impossible to relate D-PUFA experiments to routine low-cost laboratory diets often referred to as “control” diets. For a given PUFA, we therefore compare the effect of deuteration only, measuring the difference between corresponding H- and D-PUFAs. Indeed, the effect size of LPS challenge depends on many factors: age and sex, dose and route of administration, and genetic background should all have an influence. Moreover, strict quantification of morphological changes in all models of inflammation is technically challenging. For this reason, the effects of LPS are often reported as lung damage scores (for example, [10,11,12,13,14]). In studies where interalveolar septal thickness has been specifically measured, the difference between LPS-treated animals and LPS-free controls is similar to our results: LPS thickened the septa in rats and mice approximately 2.5–3-fold [15,16,17], confirming the data reported here.

**Figure 6:**
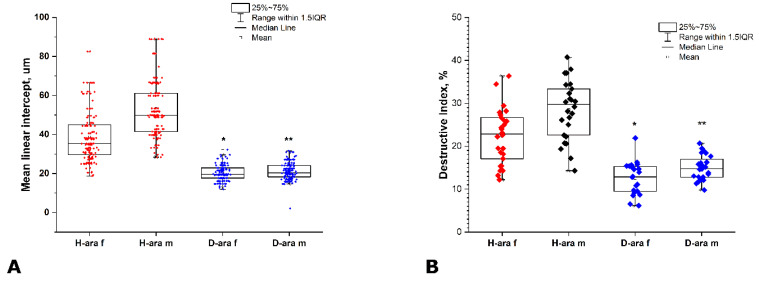
Emphysematous lumen area of female and male mice that consumed H- or D-arachidonic acid for 6 weeks and then received LPS once intranasally at a dose of 1 mg/kg. MLI (**A**) and DI (**B**) were measured as described in Materials and Methods (Section 2.4). Data are presented as means ± standard error. *—*p* ≤ 0.05 D-ARA males versus H-ARA males; **—*p* ≤ 0.05 D-ARA females versus H-ARA females.

Still, the difference between mice fed H-ARA and D6-ARA diets (Figure 5) clearly points to the existence of an in vivo isotope effect under these experimental settings. At the same time, lungs of some H-ARA mice challenged with LPS showed areas of emphysematous transformation (Figure 4, top). In addition to alveolar septal thickness, alveolar space in these emphysematous areas (Figure 4, bottom) was also quantified in LPS-treated mice (Figure 6) as mean linear intercept and destructive index. In the D6-ARA group, these parameters were found to be significantly lower than in H-ARA, indicating that this aspect of lung damage was ameliorated through deuteration of alimentary arachidonic acid.

## 4. Discussion

### 4.1. Gender-Dependent Differences

Gender differences are well known to be important in models such as ALI. For example, female mice respond more severely to *P. aeruginosa* [18]. It is believed that estrogen imparts protective effects in LPS-induced acute lung inflammation [19] and hemorrhagic shock-induced lung injury. Conversely, in infectious models that employed *P. aeruginosa*, females displayed a more robust lung inflammatory response while displaying higher bacterial loads [18]. This underscores the complexities underlying the sex differences in lung immune responses. In our study, the degree of alveolar lumen area change in both groups of animals also depended on gender: this indicator was significantly lower in females than in males, regardless of the form of ARA (H or D). Additionally, in mice on D-ARA the severity of changes in the thickness of interalveolar septa was significantly higher in males compared to females, correlating well with human cases—females being less severely affected, for example, by COVID-19 (reviewed in [20]). 

### 4.2. LPS Model of ALI/ARDS

ARDS can result from direct (e.g., pneumonia, acid aspiration) or indirect (e.g., pancreatitis, non-pulmonary sepsis) injury to the lungs. Murine models of direct ALI include those in which agents are delivered intratracheally or intranasally. We induced ALI by intranasal administration of *E. coli* LPS, a common inductor of lung injury and inflammation. Dietary consumption of H-ARA prior to LPS exposure aggravated LPS-induced lung tissue damage, including interalveolar septal thickening, while D-ARA substantially mitigated the damaging effect of LPS. LPS-induced ALI is a well-established model of ARDS [21]. LPS activates NF-κB and then the NLRP3 inflammasome, leading to secretion of IL-1β and IL-18, which further triggers inflammation by positive feedback. Although various treatments in the murine models reportedly protect from acute LPS effects, for example, ouabain [22], along with various other compounds such as xanthohumol [23] and corylin [24], the protective mechanisms are complex. In contrast, D-PUFA may have a simpler inflammation-reducing mode of action. We have previously demonstrated the inhibitory effect of bis-allylic deuteration of ARA on the formation of various pro-inflammatory eicosanoids [3] while simultaneously inhibiting LPO [3,25,26,27]. The LPS-induced lung damage model reported herein seems to validate these earlier findings.

### 4.3. D2-LIN 

Neural tissues contain ARA but not LIN, while in adipose tissue the predominant n-6 species is LIN [2]. ARA derived from LIN through the enzymatic elongation and desaturation is then incorporated into essentially every tissue. Accordingly, dosing mammals on D2-LIN would lead to D2-LIN and 13,13-D2-ARA (Figure 1) tissue incorporation [28]. Deuteration at C13 might lead to inhibition of COX1/2 as well as 15-LOX, reducing the level of some pro-inflammatory species [3] as well as LPO. Indeed, our preliminary experiments revealed that although LPS-induced changes in alveolar morphology were found in mice consuming both H- or D2-LIN for 6 weeks, the thickness of the interalveolar septum was significantly higher in mice treated with H-LIN compared to the D2-LIN group, regardless of gender (Figure 2). Consumption of D-ARA for 6 weeks led to greater protective action against LPS-induced interalveolar septal thickening (Figure 6). 

### 4.4. D6-ARA

H-ARA served as the control while D-ARA was the experimental treatment, thus isolating the bis-allylic D of ARA as the sole factor differing in the study. Our results, therefore, avoid confounding with variable fat composition of low-cost closed formula diets used routinely for mouse colony maintenance [29]. Notably, routine laboratory chow may or may not contain ARA or metabolically competing omega-3 fatty acids from, for instance, fish meal added as a source of inexpensive high-quality protein, or a variable level of natural antioxidants. The non-fat components of our diets were identical, as was the added fat, with the only difference being the ARA components, H-ARA or D6-ARA. Alimentary ARA is more efficiently incorporated into tissue than ARA derived from oral LIN. Nevertheless, within a tissue or pool (e.g., plasma phospholipids), ARA levels tend to be more tightly controlled than other long chain PUFAs. Dietary ARA increases the metabolic turnover of tissue ARA.

LPS activates phospholipase A2, leading to the release of free ARA into tissues and blood, modulating overall inflammation. Accordingly, metabolism of fatty acids is likely to be important for modulation of the acute LPS-induced lung disorder and other conditions [30]. Exogenous oxidized phospholipids induce hyperinflammatory reactions in vitro [31]. n-3 PUFAs down-regulate inflammation by competing with ARA for LOX and COX, while some PUFA oxidation products (e.g., isoprostanes and neuroprostanes) are biologically active [32,33]. A single ARA bolus resulted in an increase in superoxide dismutase (SOD), decreased myeloperoxidase (MPO) activity, reduced malondialdehyde (MDA), lowered lactate dehydrogenase (LDH), alleviated PQ-induced histological damage, and inductions of inflammatory cytokines [34]. This indicates that multiple mechanisms of acute or chronic responses exist, perhaps involving pro-resolving action of some of the ARA oxidation products, such as asthma-relevant pro-resolving mediators (SPMs) such as PGE2 and LXB4 [35]. In bacterial infections, the situation is more complex because some microorganisms, e.g., the common opportunistic pathogen *P. aeruginosa*, produce a secreted form of lipoxygenase that oxidizes ARA [36].

ARA is the major PUFA involved in inflammation as a precursor of numerous enzymes initiating the eicosanoid cascade. For example, peripheral blood lymphocytes and monocytes have been reported to have an average ARA content of 16–20% of total fatty acids. Membrane ARA released by phospholipase A2 (PLA2) acts as a substrate for COX, LOX, and cytochrome P450, yielding pro-inflammatory eicosanoid mediators and regulators, such as prostaglandins, leukotrienes, and thromboxanes. Patients with acute COPD have increased ARA as a free fatty acid in sputum. The severity of emphysematous transformations may also correlate in such patients with high ARA levels and COX2- converted mediators [37]. In our study, dietary consumption of D-ARA for 6 weeks significantly decreased endotoxin-induced peribronchial infiltration and improved the thickness of interalveolar septa as well as signs of alveolar emphysematous transformations compared relative to the mice receiving H-ARA, suggesting that (i) increased formation of eicosanoids from dietary H-ARA and (ii) successful displacement of n-6 PUFA in lung and surfactant PLs by D-ARA (more stable with respect to both enzymatic and non-enzymatic oxidation) results in decreased production of COX- and LOX-converted mediators as well as decreased PLA2 stimulation by oxidative stress [38].

### 4.5. Role of Surfactant

Pulmonary surfactant covers the large alveolar surface in all mammalian species, stabilizing the alveoli and preventing them from water tension-induced collapse. It is composed of 10% protein and 90% lipids, mostly phospholipids (PLs). Within PLs, both acyl chains can be either saturated, monounsaturated, or polyunsaturated (PUFAs) [1]. The structure of surfactant PLs permits them to modulate the host–pathogen interaction as well as to respond to environment-induced oxidative stress. Alterations of the pulmonary surfactant system have long been documented in ARDS and other lung diseases with pronounced alveolar inflammation. Large surfactant aggregates from patients with ARDS or pneumonia are characterized by a substantial decrease in palmitic acid, whereas the relative level of PUFAs (including ARA) in PLs is significantly increased [39]. Although an intra-alveolar microenvironment is often considered not to be in direct physical contact with the plasma pool, there is evidence suggesting that systemic metabolic status, as well as dietary variability, can modulate surfactant PLs. Fish oil supplementation significantly modulated the PUFA composition of rat surfactant PLs within 72 h. Thus, the n-6 PUFAs (LIN and ARA, the precursors of eicosanoids and leukotrienes) were displaced by long chain n-3 PUFAs. Such an ability to rapidly modulate the PUFA composition of surfactant may facilitate attenuation of eicosanoid-driven inflammatory processes and decrease the risk of developing ARDS. 

### 4.6. Animal Models Are Imperfect

Extrapolating underlying molecular mechanisms from mouse data to humans is complex due to known differences in COX and LOX [40]. The number of active isoforms (seven in mice and six in humans), substrate specificities [41], metabolic products [42], and the pharmacological profiles of regulating networks may differ [43]. However, at least for in vitro cultured human macrophages, LPS directly affects ARA metabolism through the release of both free ARA cyclooxygenase products [44,45], indicating that the major regulatory pathways are conserved between mammals as diverse as rodents and primates. The difference between acute and chronic exposure to LPS and other toxins [46] is also important. This is relevant to many disease models, for example, dust mite extract induced asthmatic mice ARA levels to be upregulated [47]. In asthma, ARA may be beneficial because it is the source of the specialized pro-resolving mediators (SPMs), such as PGE2 and LXB4 [35]. PUFA dysregulation in pulmonary allergic diseases is reviewed in detail elsewhere [48,49]. 

### 4.7. ARA, the Yin and Yang of Inflammation

Excessive ARA is pro-inflammatory under conditions of viral or bacterial infections, which can be modelled by LPS. LPS treatment promotes inflammation through various mechanisms, including the pro-inflammatory cytokines [50], eicosanoids [51], and induction of ROS [52]. Following the intranasal administration of LPS, ALI quickly follows. The COVID-19 pandemic is causing severe morbidity and mortality across the globe. Advanced age and other pre-existing conditions result in elevated mortality among COVID-19 patients. Many viruses have high affinity towards receptors found in the lungs. COVID-19, SARS, MERS, influenza, and some non-infectious diseases trigger an immune response, attracting immune cells to the region to attack the virus, resulting in localized inflammation [53]. In some cases, “cytokine storms” ensue, involving excessive uncontrolled production of pro-inflammatory cytokines/chemokines, including IL-6, TNF-α, IFN-γ, IL-2, and IL-7, resulting in potentially fatal hyperinflammation. The immune system of younger people is more reliant on innate mechanisms, so it usually produces lower levels of inflammation-driving cytokines. Additionally, COVID-19 patients demonstrate an increased incidence of thrombotic complications (almost half of the patients admitted to the ICU), even with thromboprophylaxis, further contributing to the high mortality rate from COVID-19 due to pulmonary embolism, strokes, and heart attacks. ARA oxidation products are elevated in COVID-19 patients [54,55,56]. 

### 4.8. Perspectives on Dietary D-ARA as a Drug

Traditional approaches to mitigating the adverse effects of eicosanoid-mediated excessive inflammatory response rely on small molecule inhibitors of COX and LOX enzymes, which can lead to worsening of pulmonary symptoms (e.g., aspirin-aggravated respiratory disease, where dysregulated ARA metabolism plays an essential role [57]). Thus, D-ARA may represent a milder approach to treatment of inflammatory lung pathologies. 

Oral data from human dosing in ongoing clinical trials show an impressive safety record for D2-LIN, a metabolic precursor to 13,13-D2-ARA [28,58,59]. The expected safety of a D-PUFA drug combined with the convenience of oral dosing of D-PUFA gel caps warrants further studies of D-PUFAs as part of an approach for anti-inflammatory, and possibly preventative, therapy against COVID-19-induced cytokine storm and thrombosis events. Other PUFA emulsions such as IntraLipid^®^ are formulated in emulsions and dosed I.V. at multiple grams per day safely. One could imagine that such a formulation could enable rapid, bolus dosing of the drug upon treatment onset, followed by lipid gel cap oral dosing of D-ARA as a continuing therapy. These data present a rationale for further research on D-PUFA technology as an approach to COVID-19 inflammation-associated therapy.

D-ARA is less prone to either enzymatic or non-enzymatic oxidation and so is more potent in regulatory interactions that do not require chemical conversions. In high-fat diet-fed mice, ARA reduces LPS-triggered inflammation in macrophages and septic death in mice through binding to MD2 [60]. Thus, in addition to its enhanced stability, D-ARA may also be beneficial due to its improved metabolism. 

In the future, D-ARA may become a viable therapeutic for inflammatory conditions involving enzymatic and non-enzymatic oxidation; however, many more mechanistic and toxicological studies are required before any clinical developments.

## Figures and Tables

**Table 1:** Fat content in mouse diets.

Fat Composition of Research Diets	H-ARA (Green Dye)	D-ARA (Pink Dye)
Saturated fat, %	7.75	7.75
High oleic sunflower, %	3.1	3.1
H-linolenic (ethyl linolenate), %	0.2	0.2
ARA ethyl ester, %	0.25 (H-ARA)	0.25 (D-ARA)

## Data Availability

Data is contained within the article.

## Supplementary Materials

The following supporting information can be downloaded at: https://www.mdpi.com/article/10.3390/antiox11040681/s1.
