# Supplementary material for: Deuterated Arachidonic Acid Ameliorates Lipopolysaccharide-Induced Lung Damage in Mice
Source: Antioxidants (Basel). 2022 Mar 31;11(4):681. doi: 10.3390/antiox11040681 (PMC9027010; doi:10.3390/antiox11040681)
Supplement: Supplementary file 1 [file antioxidants-11-00681-s001.zip › SUPPLEMENTAL DATA-Molchanova.pdf]

Alla Y. Molchanova, Svetlana N. Rjabceva, Tigran B. Melik-Kasumov, Nikolay B. Pestov, Plamena R. Angelova, Vadim V. Shmanai, Olga L. Sharko, Andrei V. Bekish, Genevieve James, Hui Gyu Park, Irina A. Udalova, J. Thomas Brenna, and Mikhail S. Shchepinov

Deuterated Arachidonic Acid Ameliorates Lung Damage in Lipopolysaccharide-treated Mice

SUPPLEMENTARY DATA

Analysis of IL1β

Supplementary Table S1. Descriptive statistics of the concentration of IL-1β in BAL fluids of male mice fed H-ARA and 6D-ARA diets. Results of ELISA test for IL-1β, (pg/ml). 2H, 2D, 4H, 4D, 6H, 6D, - number of days on diet and H-arachidonic or 6D-arachidonic acid.

	N total	Mean	SE	Min	Q1	Median	Q3	Max
control	7	0.83	0.21	0.15	0.30	0.74	1.37	1.52
2H	9	2.36	0.99	0.10	0.42	1.46	2.92	9.58
2D	9	7.55	4.60	0.63	0.73	3.02	5.73	43.60
4H	11	4.69	1.40	0.78	1.43	2.60	8.72	14.97
4D	10	2.47	0.77	0.26	0.26	2.08	3.65	7.55
6H	9	2.52	0.79	0.78	1.12	1.67	2.68	8.37
6D	10	2.43	0.41	1.00	1.34	2.23	3.35	4.91

Supplementary Table S2. Descriptive statistics of the concentration of IL-1β in BAL fluids of female mice fed H-ARA and 6D-ARA diets. Results of ELISA test for IL-1β, (pg/ml). 2H, 2D, 4H, 4D, 6H, 6D, - number of days on diet and H-arachidonic or 6D-arachidonic acid.

	N total	Mean	SE	Min	Q1	Median	Q3	Max
control	7	1.03	0.30	0.21	0.42	0.53	2.01	2.01
2H	10	0.93	0.33	0.12	0.25	0.55	0.74	3.06
2D	9	1.48	0.47	0.12	0.37	0.49	2.57	3.43
4H	10	1.69	0.39	0.52	0.78	0.98	3.13	3.65
4D	10	1.58	0.40	0.39	0.78	0.98	2.47	3.78
6H	10	3.44	1.66	0.38	0.88	1.38	3.38	17.91
6D	10	2.75	0.42	0.88	1.88	2.88	4.00	4.50

Supplementary Table S3. Descriptive statistics of the concentration of IL-1β in BAL fluids of male mice fed H-ARA and 6D-ARA diets and challenged with intranasal LPS for 24 h. Results of ELISA test for IL-1β, (pg/ml). 2H, 2D, 4H, 4D, 6H, 6D, - number of days on diet and H-arachidonic or 6D-arachidonic acid.

	N	Mean	SE	Minimum	Q1	Median	Q3	Max
control	7	0.83	0.21	0.15	0.30	0.74	1.37	1.52
control+LPS	8	1.25	0.46	0.21	0.26	1.01	1.58	4.12
2H+LPS	8	4.04	2.17	0.10	0.48	2.26	4.01	18.71
2D+LPS	9	5.92	1.72	0.63	1.77	3.33	8.57	15.05
4H+LPS	9	1.95	0.45	0.52	0.91	1.43	3.39	4.17
4D+LPS	10	15.83	6.44	0.39	2.99	5.40	20.45	54.89
6H+LPS	10	2.03	0.26	0.89	1.23	2.40	2.68	3.01
6D+LPS	9	2.74	0.59	0.33	2.01	2.46	3.13	6.92

Supplementary Table S4. Descriptive statistics of the concentration of IL-1β in BAL fluids of female mice fed H-ARA and 6D-ARA diets and challenged with intranasal LPS for 24 h. Results of ELISA test for IL-1β, (pg/ml). 2H, 2D, 4H, 4D, 6H, 6D, - number of days on diet and H-arachidonic or 6D-arachidonic acid.

	N	Mean	SE	Min	Q1	Median	Q3	Max
control	7	1.03	0.30	0.21	0.42	0.53	2.01	2.01
control+LPS	8	0.77	0.14	0.32	0.48	0.58	1.17	1.38
2H+LPS	8	4.47	3.15	0.12	0.25	2.08	2.27	26.41
2D+LPS	9	1.01	0.32	0.25	0.37	0.37	1.84	2.57
4H+LPS	10	1.32	0.64	0.13	0.39	0.52	1.30	6.77
4D+LPS	9	1.26	0.21	0.65	0.78	1.04	1.82	2.21
6H+LPS	10	2.29	0.37	1.00	1.25	2.13	2.88	4.63
6D+LPS	10	2.49	0.33	1.13	1.75	2.13	3.38	4.25

Supplementary Table S5. Descriptive statistics of the concentration of IL-1β in colon homogenates of male mice fed H-ARA and 6D-ARA diets. Results of ELISA test for IL-1β, (ng/ml). 2H, 2D, 4H, 4D, 6H, 6D, - number of days on diet and H-arachidonic or 6D-arachidonic acid.

	N	Mean	SE	Min	Q1)	Median	Q3	Max
control	8	2.74	0.26	1.98	2.22	2.56	3.08	4.25
2H	10	2.87	0.44	1.10	2.08	2.87	3.68	5.89
2D	10	2.59	0.19	1.68	2.08	2.74	2.87	3.52
4H	11	2.83	0.45	1.21	1.34	2.77	4.00	5.84
4D	10	2.85	0.54	1.72	1.96	2.11	3.10	7.44
6H	9	4.10	0.55	1.73	3.10	3.84	4.91	6.67
6D	10	3.24	0.29	1.75	2.72	3.27	4.07	4.61

Supplementary Table S6. Descriptive statistics of the concentration of IL-1β in colon homogenates of female mice fed H-ARA and 6D-ARA diets. Results of ELISA test for IL-1β, (ng/ml). 2H, 2D, 4H, 4D, 6H, 6D, - number of days on diet and H-arachidonic or 6D-arachidonic acid.

	N	Mean	SE	Min	Q1	Median	Q3)	Max
control	8	2.03	0.37	1.15	1.55	1.62	2.11	4.50
2H	10	2.81	0.37	1.46	2.24	2.63	2.90	5.85
2D	10	3.12	0.23	1.94	2.53	3.16	3.45	4.40
4H	10	3.01	0.19	1.62	2.82	3.18	3.36	3.71
4D	10	3.28	0.31	1.80	2.59	3.28	3.84	4.89
6H	10	2.62	0.22	1.37	2.27	2.61	3.09	3.57
6D	10	3.08	0.29	1.27	2.42	3.15	3.90	4.06

Supplementary Table S7. Descriptive statistics of the concentration of IL-1β in colon homogenates of male mice fed H-ARA and 6D-ARA diets and challenged with intranasal LPS for 24 h. Results of ELISA test for IL-1β, (ng/ml). 2H, 2D, 4H, 4D, 6H, 6D, - number of days on diet and H-arachidonic or 6D-arachidonic acid.

	N	Mean	SE	Min	Q1	Median	Q3	Max
control	8	2.7	0.3	2.0	2.2	2.6	3.1	4.2
control+LPS	8	2.2	0.3	1.0	1.5	2.5	2.8	3.3
2H+LPS	10	1.8	0.3	1.0	1.1	1.5	2.5	3.6
2D+LPS	10	2.7	0.3	1.7	2.2	2.4	3.1	4.8
4H+LPS	9	2.6	0.6	1.4	1.7	2.2	2.5	7.0
4D+LPS	10	1.9	0.2	0.6	1.4	2.0	2.6	2.8
6H+LPS	10	2.6	0.2	1.9	2.2	2.6	3.0	3.7
6D+LPS	10	2.5	0.4	1.2	1.7	2.2	3.2	5.0

Supplementary Table S8. Descriptive statistics of the concentration of IL-1β in colon homogenates of female mice fed H-ARA and 6D-ARA diets and challenged with intranasal LPS for 24 h. Results of ELISA test for IL-1β, (ng/ml). 2H, 2D, 4H, 4D, 6H, 6D, - number of days on diet and H-arachidonic or 6D-arachidonic acid.

	N	Mean	SE	Min	Q1	Median	Q3	Max
control	8	2.03	0.37	1.15	1.55	1.62	2.11	4.50
control+LPS	8	1.91	0.39	0.80	1.12	1.55	2.58	3.97
2H+LPS	9	2.67	0.44	0.62	2.16	2.72	2.88	5.10
2D+LPS	9	2.53	0.40	1.45	1.83	2.07	3.09	5.21
4H+LPS	10	1.95	0.19	0.96	1.49	1.94	2.39	2.97
4D+LPS	9	2.10	0.25	1.33	1.70	1.76	2.48	3.68
6H+LPS	10	2.21	0.18	1.25	1.97	2.24	2.46	3.27
6D+LPS	10	2.09	0.18	1.42	1.61	2.04	2.40	3.21

Supplementary Table S9. Descriptive statistics of the number of lymphocytes in BAL fluids of male mice fed H-ARA and 6D-ARA diets. 2H, 2D, 4H, 4D, 6H, 6D, - number of days on diet and H-arachidonic or 6D-arachidonic acid.

	N t	Mean	SE	Min	Q1)	Median	Q3	Max
control	8	0,625	0,183	0	0	1	1	1
2H	10	4,8	1,645	0	1	2,5	8	15
2D	10	2,9	0,657	1	2	2	3	8
4H	10	9,1	5,496	1	2	3	5	58
4D	10	7,1	2,541	1	2	4	8	25
6H	9	6,667	1,871	1	2	5	10	15
6D	10	2,4	0,4	1	2	2	3	5

Supplementary Table S10. Descriptive statistics of the number of lymphocytes in BAL fluids of female mice fed H-ARA and 6D-ARA diets. 2H, 2D, 4H, 4D, 6H, 6D, - number of days on diet and H-arachidonic or 6D-arachidonic acid.

	N total	Mean	SE	Min	Q1)	Median	Q3	Max
control	8	0.625	0.183	0	0	1	1	1
2H	10	1.3	0.396	0	0	1.5	2	3
2D	10	1.5	0.307	0	1	2	2	3
4H	10	3.1	0.640	0	2	2.5	5	6
4D	10	10.4	3.961	3	4	6	11	45
6H	10	4	0.558	2	2	4	5	7
6D	10	7	1.549	1	3	6	12	15

Supplementary Table S11. Descriptive statistics of the number of neutrophils in BAL fluids of male mice fed H-ARA and 6D-ARA diets. 2H, 2D, 4H, 4D, 6H, 6D, - number of days on diet and H-arachidonic or 6D-arachidonic acid.

	N	Mean	SE	Min	Q1	Median	Q3	Max
control	8	0.125	0.125	0	0	0	0	1
2H	10	0.6	0.499	0	0	0	0	5
2D	10	1.4	0.733	0	0	0	3	7
4H	10	1.9	1.224	0	0	0	1	11
4D	10	1	0.683	0	0	0	0	6
6H	9	1.333	0.667	0	0	1	1	6
6D	10	0.9	0.379	0	0	1	1	4

Supplementary Table S12. Descriptive statistics of the number of neutrophils in BAL fluids of female mice fed H-ARA and 6D-ARA diets. 2H, 2D, 4H, 4D, 6H, 6D, - number of days on diet and H-arachidonic or 6D-arachidonic acid.

	N	Mean	SE	Min	Q1	Median	Q3	Max
control	8	0.25	0.164	0	0	0	0.5	1
2H	10	0.6	0.4	0	0	0	1	4
2D	10	0.7	0.3	0	0	0.5	1	3
4H	10	0.4	0.163	0	0	0	1	1
4D	10	0.1	0.1	0	0	0	0	1
6H	10	1.6	0.75	0	0	1	2	8
6D	10	1.2	0.39	0	0	1	2	4

Supplementary Table S13. Descriptive statistics of the number of plasmacytes in BAL fluids of male mice fed H-ARA and 6D-ARA diets. 2H, 2D, 4H, 4D, 6H, 6D, - number of days on diet and H-arachidonic or 6D-arachidonic acid.

	N	Mean	SE	Min	Q1)	Median	Q3)	Max
control	8	0,25	0,164	0	0	0	0,5	1
2H	10	0,7	0,335	0	0	0	1	3
2D	10	0,9	0,314	0	0	1	1	3
4H	10	0,7	0,260	0	0	0,5	1	2
4D	10	0,8	0,2	0	0	1	1	2
6H	9	0,56	0,242	0	0	0	1	2
6D	10	0,3	0,153	0	0	0	1	1

Supplementary Table S14. Descriptive statistics of the number of plasmacytes in BAL fluids of female mice fed H-ARA and 6D-ARA diets. 2H, 2D, 4H, 4D, 6H, 6D, - number of days on diet and H-arachidonic or 6D-arachidonic acid.

	N	Mean	SE	Min	Q1	Median	Q3	Max
control	8	0.125	0.125	0	0	0	0	1
2H	10	0.8	0.291	0	0	1	1	3
2D	10	0.4	0.163	0	0	0	1	1
4H	10	0.5	0.167	0	0	0.5	1	1
4D	10	0.8	0.133	0	1	1	1	1
6H	10	0.6	0.163	0	0	1	1	1
6D	10	0.8	0.249	0	0	1	1	2

Supplementary Table S15. Descriptive statistics of the number of macrophages in BAL fluids of male mice fed H-ARA and 6D-ARA diets. 2H, 2D, 4H, 4D, 6H, 6D, - number of days on diet and H-arachidonic or 6D-arachidonic acid.

	N	Mean	SE	Min	Q1	Median	Q3	Max
control	8	0.75	0.366	0	0	0.5	1	3
2H	10	1.9	0.482	0	1	1.5	2	5
2D	10	1.1	0.277	0	0	1	2	2
4H	10	1.2	0.249	0	1	1	1	3
4D	10	1.4	0.267	0	1	1	2	3
6H	9	1.78	0.278	1	1	2	2	3
6D	10	1.6	0.340	0	1	1.5	2	4

Supplementary Table S16. Descriptive statistics of the number of macrophages in BAL fluids of female mice fed H-ARA and 6D-ARA diets. 2H, 2D, 4H, 4D, 6H, 6D, - number of days on diet and H-arachidonic or 6D-arachidonic acid.

	N	Mean	SE	Min	Q1	Median	Q3	Max
control	8	0.375	0.18298	0	0	0	1	1
2H	10	1.5	0.22361	1	1	1	2	3
2D	10	1.1	0.23333	0	1	1	2	2
4H	10	1.1	0.17951	0	1	1	1	2
4D	10	1.3	0.15275	1	1	1	2	2
6H	10	1.8	0.24944	1	1	2	2	3
6D	10	2.5	0.45338	1	2	2	3	5

Supplementary Table S17. Descriptive statistics of the number of lymphocytes in BAL fluids of male mice fed H-ARA and 6D-ARA diets and challenged with intranasal LPS for 24 h. 2H, 2D, 4H, 4D, 6H, 6D, - number of days on diet and H-arachidonic or 6D-arachidonic acid.

	N	Mean	SE	Min	Q1	Median	Q3	Max
control	8	0.625	0.183	0	0	1	1	1
control+LPS	8	0.56	0.176	0	0	1	1	1
2H+LPS	10	1.2	0.359	0	0	1	2	3
2D+LPS	10	1.2	0.249	0	1	1	1	3
4H+LPS	10	4.4	1.492	0	1	3.5	7	15
4D+LPS	10	10.6	4.564	1	2	4	15	45
6H+LPS	10	3.6	0.819	1	2	2.5	5	9
6D+LPS	9	5.22	1.289	0	2	4	9	11

Supplementary Table S18. Descriptive statistics of the number of lymphocytes in BAL fluids of female mice fed H-ARA and 6D-ARA diets and challenged with intranasal LPS for 24 h. 2H, 2D, 4H, 4D, 6H, 6D, - number of days on diet and H-arachidonic or 6D-arachidonic acid.

	Mean	SE	Min	Q1	Median	Q3	Max
control	3.2	0.2	0.7	2.6	3.2	3.9	5.3
control+LPS	6.3	0.5	1.4	4.1	5.4	7.9	13.5
2H+LPS	5.2	0.2	2.6	4.1	5.0	5.9	11.2
2D+LPS	6.4	0.4	1.8	4.4	5.5	8.4	16.3
4H+LPS	4.7	0.3	2.2	3.3	4.5	5.4	11.7
4D+LPS	4.6	0.3	1.6	3.3	4.1	5.1	13.6
6H+LPS	7.1	0.3	3.6	5.4	7.1	8.0	15.9
6D+LPS	5.1	0.3	1.8	3.4	5.2	6.1	9.3

Supplementary Table S19. Descriptive statistics of the number of neutrophils in BAL fluids of male mice fed H-ARA and 6D-ARA diets and challenged with intranasal LPS for 24 h. 2H, 2D, 4H, 4D, 6H, 6D, - number of days on diet and H-arachidonic or 6D-arachidonic acid.

	N	Mean	SE	Min	Q1	Median	Q3	Max
control	8	0.125	0.125	0	0	0	0	1
control+LPS	8	1.625	0.375	1	1	1	2	4
2H+LPS	10	5.8	2.69	1	1	1.5	7	28
2D+LPS	10	21.7	16.39	0	1	4	11	168
4H+LPS	10	7.2	1.356	0	6	7	12	12
4D+LPS	10	16.3	8.912	0	2	4.5	11	87
6H+LPS	10	2.8	0.66	0	1	2.5	5	6
6D+LPS	9	2.56	0.85	0	2	2	2	9

Supplementary Table S20. Descriptive statistics of the number of neutrophils in BAL fluids of female mice fed H-ARA and 6D-ARA diets and challenged with intranasal LPS for 24 h. 2H, 2D, 4H, 4D, 6H, 6D, - number of days on diet and H-arachidonic or 6D-arachidonic acid.

	N	Mean	SE	Min	Q1	Median	Q3	Max
control	8	0.25	0.164	0	0	0	0.5	1
control+LPS	8	5.375	2.520	1	1	3	6	22
2H+LPS	9	21.22	15.245	1	1	2	9	140
2D+LPS	9	11.89	4.581	1	4	7	19	43
4H+LPS	10	10.9	4.574	1	2	3.5	21	45
4D+LPS	10	8.7	3.493	1	1	4	11	32
6H+LPS	10	5.7	1.633	1	2	3.5	9	16
6D+LPS	10	5.3	1.850	1	2	3.5	6	21

Supplementary Table S21. Descriptive statistics of the number of plasmacytes in BAL fluids of male mice fed H-ARA and 6D-ARA diets and challenged with intranasal LPS for 24 h. 2H, 2D, 4H, 4D, 6H, 6D, - number of days on diet and H-arachidonic or 6D-arachidonic acid.

	N	Mean	SE	Min	Q1	Median	Q3	Max
control	8	0.25	0.163	0	0	0	0.5	1
control+LPS	8	0.625	0.182	0	0	1	1	1
2H+LPS	10	1.2	0.36	0	0	1	2	3
2D+LPS	10	1.2	0.249	0	1	1	1	3
4H+LPS	10	1.2	0.327	0	1	1	1	3
4D+LPS	10	1.2	0.327	0	0	1	2	3
6H+LPS	10	0.9	0.233	0	0	1	1	2
6D+LPS	9	0.11	0.111	0	0	0	0	1

Supplementary Table S22. Descriptive statistics of the number of plasmacytes in BAL fluids of female mice fed H-ARA and 6D-ARA diets and challenged with intranasal LPS for 24 h. 2H, 2D, 4H, 4D, 6H, 6D, - number of days on diet and H-arachidonic or 6D-arachidonic acid.

	N total	Mean	SE	Min	Q1	Median	Q3	Max
control	8	0.125	0.125	0	0	0	0	1
control+LPS	8	0.25	0.164	0	0	0	0.5	1
2H+LPS	9	1.222	0.434	0	0	1	2	3
2D+LPS	9	0.556	0.242	0	0	0	1	2
4H+LPS	10	0.6	0.163	0	0	1	1	1
4D+LPS	9	1	0.236	0	1	1	1	2
6H+LPS	10	0.6	0.163	0	0	1	1	1
6D+LPS	10	0.2	0.133	0	0	0	0	1

Supplementary Table S23. Descriptive statistics of the number of macrophages in BAL fluids of male mice fed H-ARA and 6D-ARA diets and challenged with intranasal LPS for 24 h. 2H, 2D, 4H, 4D, 6H, 6D, - number of days on diet and H-arachidonic or 6D-arachidonic acid.

	N	Mean	SE	Min	Q1	Median	Q3	Max
control	8	0.75	0.37	0	0	0.5	1	3
control+LPS	8	0.375	0.18	0	0	0	1	1
2H+LPS	10	1.4	0.45	0	1	1	2	5
2D+LPS	10	1.8	0.39	1	1	1.5	2	5
4H+LPS	10	1.6	0.34	0	1	2	2	3
4D+LPS	10	1.7	0.40	1	1	1	2	5
6H+LPS	10	1.5	0.34	0	1	1	3	3
6D+LPS	9	1.22	0.22	0	1	1	2	2

Supplementary Table S24 Descriptive statistics of the number of macrophages in BAL fluids of female mice fed H-ARA and 6D-ARA diets and challenged with intranasal LPS for 24 h. 2H, 2D, 4H, 4D, 6H, 6D, - number of days on diet and H-arachidonic or 6D-arachidonic acid.

	N total	Mean	SE	Min	Q1	Median	Q3	Max
control	8	0.375	0.182	0	0	0	1	1
control+LPS	8	0.375	0.182	0	0	0	1	1
2H+LPS	9	1.56	0.47	0	0	2	2	4
2D+LPS	9	1.78	0.57	1	1	1	1	6
4H+LPS	10	1.6	0.21	1	1	1.5	2	3
4D+LPS	9	1.89	0.26	1	1	2	2	3
6H+LPS	10	1.6	0.3	0	1	2	2	3
6D+LPS	10	1.9	0.4	1	1	2	2	5
