# Supplementary material for: Deuterated Arachidonic Acid Ameliorates Lipopolysaccharide-Induced Lung Damage in Mice
Source: Antioxidants (Basel). 2022 Mar 31;11(4):681. doi: 10.3390/antiox11040681 (PMC9027010; doi:10.3390/antiox11040681)
Supplement: Supplementary file 1 [file antioxidants-11-00681-s001.zip › SUPPLEMENTAL FIGURE - Molchanova.pdf]

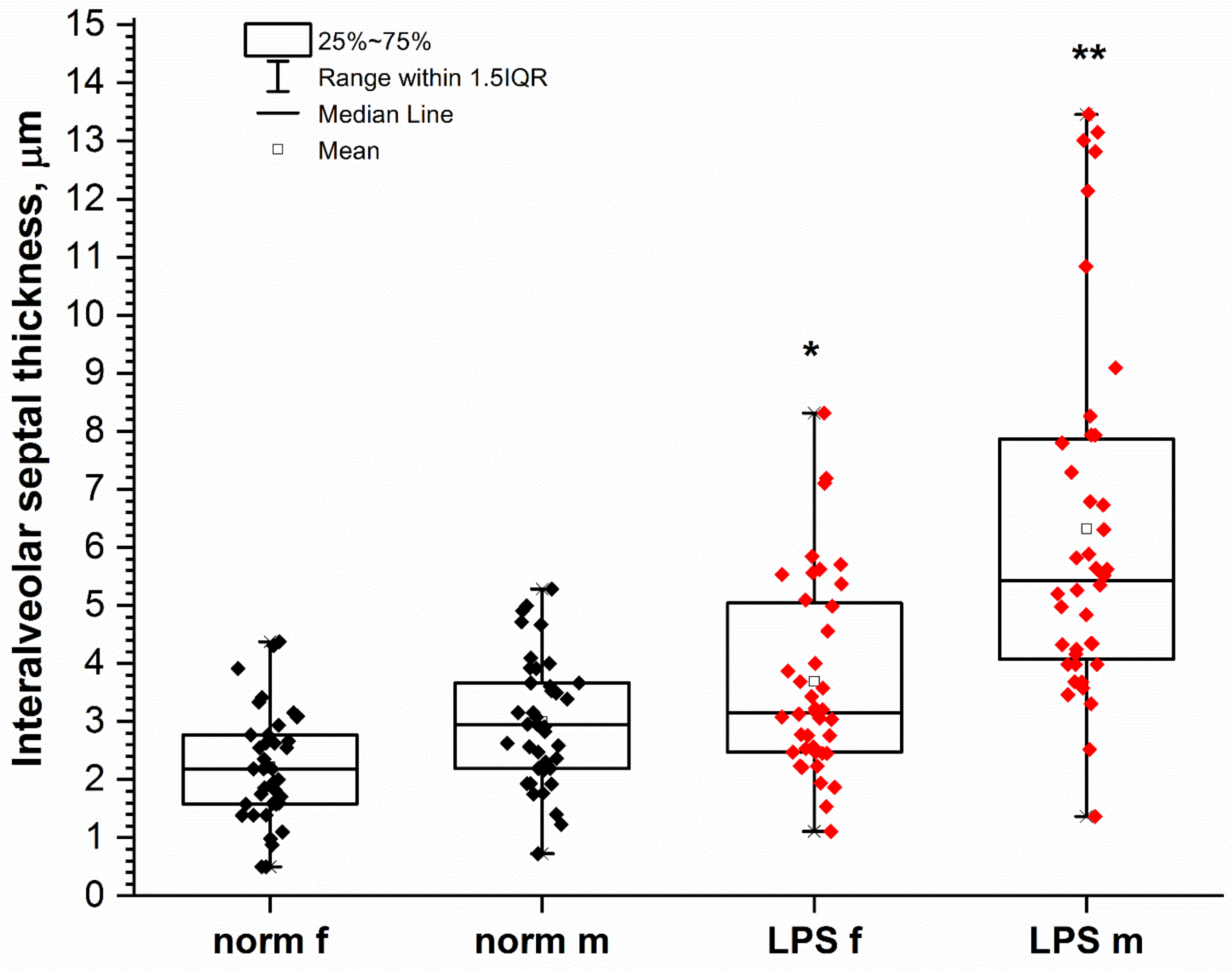

Molchanova *et al.*

Supplemental Figure S1. Interalveolar septal thickness (µм) of sexed (f –females, m – males) mice fed routine low cost diet before (norm) and after (24 h) single intranasal administration of lipopolysaccharide (LPS). * - p≤0.05, LPS treated females compared versus normal females, ** - p≤0.05, LPS treated males versus D2-Lin mice.
